# The Chemistry of Plaster of Paris

**Published:** 1906-01-15

**Authors:** William J. Manning

**Affiliations:** Washington, D. C.


### THE CHEMISTRY OF PLASTER OF PARIS.

BY WILLIAM J. MANNING, M.D., D.D.S., WASHINGTON, D. C.

Gypsum, or plaster, is found in its natural state. The chemical formula is Ca-SO4-2H2-O, it contains sulphuric acid, 46.5 percent; lime, 32.6 percent; water, 20.9 percent, according to the dictionary of mechanical arts. Its specific gravity is 2.31. The anhydride is a mineral that resembles plaster very much, but, as its name indicates, contains no water, and is composed of 58.8 percent of sulphuric acid and 41.2 percent of lime. This absorbs water and changes to plaster.

When gypsum is properly heated and calcined it loses part of its water of crystallization and becomes plaster of paris. It obtained this name from the fact that there are large deposits of gypsum found at Montmartre, a suburb of Paris. The transparent crystallized variety of gypsum is called selenite; its fine massive variety, alabaster; and its fibrous form, satin spar. There is a variety of loose, disintegrated plaster found in Kansas and other western points that is known as Gypsite. Large deposits of gypsum are found in the United States, but in trade the fact that they are located far from seaports and railways, makes the transportation and cost excessive and unable to compete with foreign material, although something like two million dollars. worth of the rock was mined in the United States in 1903. The color, too, is not as good as that obtained in Canada. Most of the plaster used for dental purposes comes from Nova Scotia and New Brunswick and is laid down in New York for something under $2.00 a ton in the crude state.

Most of the gypsum deposits of the world are considered to have been formed by the concentration and evaporation of sea water.

Sea water, according to the analyses in the *Challenger Reports* and quoted by the United States Geological Survey Reports* contains 3.5 percent of mineral matter of the following constituents:

| | |
|---|---|
| Sodium chlorid (salt) | 77.758 |
| Magnesium chlorid | 10.878 |
| Magnesium suplhate | 4.737 |
| Calcium sulphate (gypsum) | 3.600 |
| Pottassium sulphate | 2.465 |
| Calcium carbonate | 0.345 |
| Magnesium bromid | 0.217 |
| | 100.000 |

*The United States Geological Survey Reports, "The Mineral Wealth of the United States."

The calcium sulphate, however, is not precipitated until about 80 percent of the water has evaporated. When such a body of water is cut off from the ocean and evaporated the calcium sulphate is deposited before the sodium chlorid, which is not thrown down until 93 percent of the water is evaporated. Gypsum deposits are therefore more widely distributed than salt, but in thinner beds. When sulphuric acid is liberated by the decomposition of pyrite, it acts on calcium carbonate and converts it into calcium sulphate, and there is a gradual transition from the limestone to the gypsum rock.

The use of gypsum was known at a very early period. The Greeks were familiar with it, as shown by the writings of Theophrastus and Pliny. Virgil also wrote concerning the value of gypsum on cultivated lands.

As the readers of this article will be more interested in the use of plaster of Paris, as used by dentists and surgeons, it will be upon this product of gypsum that we will dwell to some extent.

Lavoisier seems to be the first man who made a study of the phenomena in the "setting" of plaster of Paris. In the *Compes Rendus* of February 17, 1765, appears the following: "After having removed the water of hydration from gypsum by heat, if it be presented to it again (this is commonly known as the mixing or tempering of plaster), it takes it back with avidity, and suddenly assumes a state of irregular cyrstallization; the small crystals which form become very confused with each other, the result of being a very hard mass." Lavoisier also discovered that when plaster was heated steadily and evenly, its water of crystallization was removed at two different stages. Payen, in 1830, found that gypsum began to lose its water of crystallization at 115 degrees C. He concluded that at a temperature of from 110 degrees to 120 degrees C was the best for calcining purposes.

Le Chatelier, in 1887, found "that on heating gypsum there were two breaks, one halt was made at 128 degrees C

and the other at 163 degrees C." This, he says, "shows that there are two hydrates given off at different temperatures."

The burning of gypsum is a very important matter, therefore, and the calciners become very expert. If it is underburned one of the hydrates is not set free, it being very essential that both hydrates are given off or the plaster is rendered useless.

Later on, Lavoisier gave his theory of the "set" of plaster: "I took the calcined plaster, which readily hardens with water, and threw it into considerable amount of water. Each molecule of plaster, in passing through the liquid, seized its molecule of water of crystallization and fell to the bottom in the form of small, brilliant needles, visible only with a strong lens. These needles dried in the open air with the aid of a moderate heat, are very soft and silky to the touch. Under a microscope it is perceived that what was taken under the lens for needles are also parallelopipeds, very fine, some thicker, many thinner and many more elongated. The plaster in this state is not capable of uniting with water, but if recalcined these small crystals lose their water of crystallization and become again true plaster, as perfect as before."

The late John Manning, after experimenting with plaster of paris for over thirty years, and an authority on the subject, confirmed the investigations of Le Chatelier and Lavoisier.

A microscopic examination conducted by the University Geological Survey of Kansas shows that ground gypsum before calcination consists in large masses of various sizes which consist of more or less broken crystals. After the plaster is calcined these masses are broken up into crystals of very much smaller size. This is accounted for from the fact that as the material is heated the water of crystallization is changed into steam and, expanding, breaks the crystals into still smaller particles. There is thus a physical change as well as a chemical one. Uncalcined gypsum, when treated with water, shows very little change.

When the dentist adds plaster to water and makes his

"batter," small, needle-like crystals are seen forming and shooting out in various directions, and these, as they become more abundant, unite and form a solid mass in which the various crystals can scarcely be distinguished. Open spaces left in the mass are finally closed in, and a firm, solid mass is the result. Crystallization is aided by the small size of the grains and the smaller grained plasters set more rapidly than the coarser grained ones, although the latter make the strongest casts for dental purposes.

During the manufacture of plaster of paris the gypsum rock, in pieces weighing from forty to fifty pounds, just as it is blasted in the quarry, is broken into pieces the size of a butternut by a breaker with a cam-like movement, and is then crushed and ground into flour in a buhr mill, a roller mill or a disintegrator. The powder is then carried into storage bins by belt buckets. From the bins the ground plaster is carried in the same manner to the calcining kettle into which it is slowly allowed to enter. The kettle is kept at 212 degrees F. for an hour and a half. The whole mass is kept in motion by a stirrer operated above by a belt and pulley. As the boiling increases the plaster is thrown out sometimes in "waves," running like so much water. This is due to the escape of the water of crystallization in the form of steam. The boiling takes about three hours; when the temperature reaches 270 degrees F. the plaster "settles down" somewhat and remains quiet. This marks the escape of the first hydrate. Soon the mass begins to boil again and so continues until a temperature of nearly 350 degrees F. is reached, when the second hydrate is given off. This usually is sufficient for the charge, and the material is run off into bins, where, after cooling, it is placed in barrels or bags for shipment.

Plaster of paris when mixed with water "sets" in from six to ten minutes. Various agents are used to hold in check this set. These substances are known as retarders. Glue has been used extensively for this purpose. Citric acid has been used, but on account of the expense involved it has fallen into disuse. Blending magnesian limes with the calcareous limes sometimes acts as a retarder, but it is uncertain. Substances of an organic nature, no matter what the form may be (bones, hair, etc), have been used and are used as the base in modern retarders. It would seem that any material of a non-crystalline nature, which when added to the water or plaster itself tends to keep the molecule apart, will delay the crystallization and retard the setting properties.

It is a well-known fact that when a salt crystal is dropped into a supersaturated solution of a salt, as per instance, sodium suplhate, the whole mass starts at once to crystallize. This is what happens when a pinch of sodium chloride is added to a saturated solution or "batter" of calcium sulphate —plaster of paris. The added salt—whether it be sodium chloride, borax, alum, etc., (such agents are known as accelerators)—starts the process of setting or crystallization almost at once in the dehydrated gypsum, and is the one point so much appreciated by dentists and the patient when plaster impressions are taken of the mouth.—*Dental Brief.*

